# IFSO Consensus on Definitions and Clinical Practice Guidelines for Obesity Management—an International Delphi Study

**DOI:** 10.1007/s11695-023-06913-8

**Published:** 2023-11-24

**Authors:** Paulina Salminen, Lilian Kow, Ali Aminian, Lee M. Kaplan, Abdelrahman Nimeri, Gerhard Prager, Estuardo Behrens, Kevin P. White, Scott Shikora, IFSO Experts Panel, Barham K. Abu Dayyeh, Nasreen Alfaris, Aayeed Al Qahtani, Barbara Andersen, Luigi Angrisani, Ahmad Bashir, Rachel L. Batterham, Estuardo Behrens, Mohit Bhandari, Dale Bond, Jean-Marc Chevallier, Ricardo V. Cohen, Dror Dicker, Claudia K. Fox, Pierre Garneau, Khaled Gawdat, Ashraf Haddad, Jacqués Himpens, Thomas Inge, Marina Kurian, Silvia Leite Faria, Guilherme Macedo, Alexander Dimitri Miras, Violeta Moize, Francois Pattou, Luis Poggi, Jaime Ponce, Almino Ramos, Francesco Rubino, Andrés Sanchez-Pernaute, David Sarwer, Arya M. Sharma, Christine Stier, Christopher Thompson, Josep Vidal, Tarissa Beatrice Zanata Petry

**Affiliations:** 1 Department of Surgery, University of Turku, Turku, Finland; 2 Division of Digestive Surgery and Urology, Turku University Hospital, P.O. Box 52, 20521 Turku, Finland; 3 Department of Surgery, Flinders University, Adelaide, SA Australia; 4 Department of General Surgery, Cleveland Clinic, Bariatric and Metabolic Institute, Cleveland, OH USA; 5 Section On Obesity Medicine, Geisel School of Medicine at Darthmouth, Hanover, NH USA; 6 Division of General & GI Surgery, Brigham & Women’s Hospital, Harvard Medical School, Boston, MA USA; 7 Division of Visceral Surgery, Vienna Medical University, Vienna, Austria; 8 New Life Center, Guatemala City, Guatemala; 9 ScienceRight International Health Research, London, ON Canada; 10 Department of Gastroenterology & Hepatology, Mayo Clinic, Rochester, MN USA; 11 Obesity Endocrine and Metabolism Center King Fahad Medical City, Riyadh, Kingdom of Saudi Arabia; 12 New You Medical Center, Riyadh, Saudi Arabia; 13 Austrian Obesity Alliance, Vienna, 1070 Austria; 14 University of Naples “Federico II” Department of Public Health, Naples, Italy; 15 GBMC at Jordan Hospital, Amman, Jordan; 16 Centre for Obesity Research University College London, University College London Hospital NHS Foundation Trust, London, UK; 17 New Life Center, Guatemala City, Guatemala; 18 Mohak Bariatrics & Robotics, Indore, Madhya Pradesh India; 19 Departments of Surgery and Research Hartford Hospital/HealthCare, Hartford, CT USA; 20 Hôpital Européen Georges Pompidou, University of Paris, Paris, France; 21 Center for the Treatment of Obesity and Diabetes, Hospital Alemao Oswaldo Cruz, São Paulo, Brazil; 22 Internal Medicine D & Obesity Clinic, Hasharon Hospital-Rabin Medical Center, Faculty of Medicine, Tel Aviv University, Tel Aviv, Israel; 23 University of Minnesota, Medical School Center for Pediatric Obesity Medicine, Minneapolis, MN USA; 24 Hôpital du Sacré-Coeur de Montréal, Montréal, Canada; 25 Ain-Shams School of Medicine, Cairo, Egypt; 26 Gastrointestinal, Bariatric, and Metabolic Center (GBMC), Amman, Jordan; 27 Department of Visceral Surgery Delta CHIREC Hospital, Brussels, Belgium; 28 Lurie Children’s Hospital of Chicago, Chicago, IL USA; 29 NYU Langone Health, New York, NY USA; 30 Gastrocirurgia de Brasília, Brasilia, Brazil; 31 Centro Hospitalar de São João, Gastroenterology and Hepatology, Porto, Portugal; 32 School of medicine, Ulster university, Derry, UK; 33 Unit of Obesity, Department of Endocrinology Hospital Clinic, Barcelona, Spain; 34 Department of Endocrine and Metabolic surgery, CHU Lille, University of Lille, Inserm, France; 35 Clinica Anglo Americana, Lima, Peru; 36 Metabolic and Bariatric Care CHI Memorial Hospital, Chattanooga, TN USA; 37 GastroObesoCenter Institute—Advanced Institute for Metabolic Optimization, Sao Paulo, Brazil; 38 School of Cardiovascular and Metabolic Medicine and Sciences, King’s College, London, UK; 39 Department of Surgery Complutense University of Madrid, Madrid, Spain; 40 Center for Obesity Research and Education at the College of Public Health at Temple University, Philadelphia, PA USA; 41 Department of Medicine, University of Alberta, Edmonton, AB Canada; 42 Division of Interdisciplinary Endoscopy, and Clinic for General and Visceral Surgery, Mannheim University Hospital, Mannheim, Germany; 43 Division of Gastroenterology, Hepatology and Endoscopy Brigham and Women’s Hospital, Harvard Medical School, Boston, MA USA; 44 Obesity Unit Department of Endocrinology, Hospital Clínic, Barcelona, Spain; 45 Center for the Treatment of Obesity and Diabetes, Hospital Alemao Oswaldo Cruz, São Paulo, Brazil

**Keywords:** Obesity, Severe obesity, Metabolic bariatric surgery, Bariatric surgery, Bariatric endoscopy, Anti-obesity medications, Medical treatment, Definitions, Outcomes, Consensus, IFSO, Delphi survey

## Abstract

**Introduction:**

This survey of international experts in obesity management was conducted to achieve consensus on standardized definitions and to identify areas of consensus and non-consensus in metabolic bariatric surgery (MBS) to assist in an algorithm of clinical practice guidelines for the management of obesity.

**Methods:**

A three-round Delphi survey with 136 statements was conducted by 43 experts in obesity management comprising 26 bariatric surgeons, 4 endoscopists, 8 endocrinologists, 2 nutritionists, 2 counsellors, an internist, and a pediatrician spanning six continents over a 2-day meeting in Hamburg, Germany. To reduce bias, voting was unanimous, and the statements were neither favorable nor unfavorable to the issue voted or evenly balanced between favorable and unfavorable. Consensus was defined as ≥ 70% inter-voter agreement.

**Results:**

Consensus was reached on all 15 essential definitional and reporting statements, including initial suboptimal clinical response, baseline weight, recurrent weight gain, conversion, and revision surgery. Consensus was reached on 95/121 statements on the type of surgical procedures favoring Roux-en-Y gastric bypass, sleeve gastrectomy, and endoscopic sleeve gastroplasty. Moderate consensus was reached for sleeve gastrectomy single-anastomosis duodenoileostomy and none on the role of intra-gastric balloons. Consensus was reached for MBS in patients > 65 and < 18 years old, with a BMI > 50 kg/m^2^, and with various obesity-related complications such as type 2 diabetes, liver, and kidney disease.

**Conclusions:**

In this survey of 43 multi-disciplinary experts, consensus was reached on standardized definitions and reporting standards applicable to the whole medical community. An algorithm for treating patients with obesity was explored utilizing a thoughtful multimodal approach.

**Graphical Abstract:**

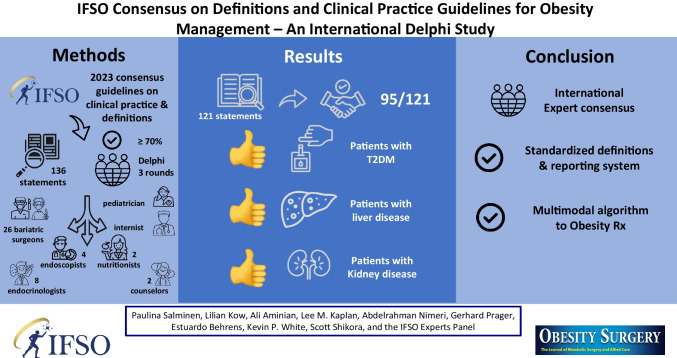

**Supplementary Information:**

The online version contains supplementary material available at 10.1007/s11695-023-06913-8.

## Introduction

The prevalence of obesity (BMI ≥ 30 kg/m^2^) is anticipated to rise from 14% in 2020 to 24% over the next 15 years and hence predicted to affect nearly 2 billion adults, children, and adolescents by 2035 [1]. The rapid rise of obesity in children and adolescents is especially concerning, since obesity in adolescence typically persists into adulthood and predisposes individuals to numerous complications [2]. Most notable obesity-associated comorbidities and complications include type 2 diabetes mellitus (T2DM) [3], cardiovascular disease [4], obstructive sleep apnea (OSA) [5], increased risks of various cancers and mortality [6, 7], reduced quality of life (QOL) [8], and increased risk of death [9–11].

Obesity is a highly heterogeneous and progressive multifactorial disease [12]. Metabolic and bariatric surgery (MBS) is the most effective treatment for severe obesity, generating substantial, sustained weight reduction, along with improvements in comorbidities and quality of life, and increased life expectancy [10, 11]. Although MBS is the most effective anti-obesity intervention, there are large variations in treatment response after MBS [13, 14], mainly due to the heterogeneity of the disease. New anti-obesity medications (AOMs) and endoscopic bariatric procedures are extremely welcome additions to the treatment of obesity linked to promising weight loss and favorable associated metabolic changes, in selected patients [15]. With the increased availability of potent AOMs now and in the near future, the practice of combination therapy will grow as MBS and AOMs can work in synergy on the treatment of severe obesity and hopefully in enabling increased access to effective obesity treatments.

In addition to the two most common surgical procedures—sleeve gastrectomy (SG) and Roux-en-Y gastric bypass (RYGB)—there are numerous other MBS procedures and considerable variation between practices and regions [16]. Variations in reported MBS outcomes (e.g., lack of uniform standardized reporting definitions) are evident in the MBS literature and markedly limit the comparability of different studies, creating a major hindrance to evidence-based clinical obesity treatment algorithms. In 2015, the American Society for Metabolic and Bariatric Surgery (ASMBS) published reporting standards in MBS aiming to enhance the quality and comparability of MBS results [17]. However, only four studies published from 2015 to 2020 have used the recommended ASMBS reporting standards, resulting in low compliance and implementation of such standards in clinical practice [18]. This highlights the importance of having valid, simple-to-use definitions for both clinical practice and research that are acceptable and applicable to the surgical and medical communities.

The aim of this consensus meeting and Delphi survey of international experts in obesity management was to achieve consensus on standardized uniform reporting definitions and standards for the whole medical community to assist in developing clinical practice guidelines for the management of severe obesity and particularly for MBS. Specific aims were to identify areas of expert consensus to assist with algorithm development, combined with a thorough review of published literature, and to identify areas of non-consensus to flag topics warranting further research.

## Methods

A three-round Delphi survey of 43 intercontinental, interdisciplinary experts in obesity management was conducted, beginning with in-person voting over 2 days in Hamburg, Germany, from March 9 to 10, 2023, followed by discussion and two rounds of online voting. The invited expert panel included bariatric surgeons, pediatric bariatric surgeons, bariatric endoscopists, endocrinologists, pediatricians, dieticians, psychologists, and counsellors with obesity management expertise. To be considered for the expert panel, clinicians had to have obesity management as a major focus of their practice, be considered experts by IFSO, have ≥ 10 years’ experience managing patients with obesity, be fluent in both spoken and written English, and be willing to attend, preferably in person, a 2-day conference in Hamburg for expert lectures on published evidence-based literature with graded-level evidence, open discussion, and a Delphi survey.

### Survey Development

In January 2023, each expert participant was asked to contribute 3–5 statements within their field for consideration by a core advisory group comprised of IFSO members and an MD-PhD level expert in Delphi surveys. This yielded > 300 submitted statements. Over six virtual meetings of the core advisory group, these were pared down to 136 statements subdivided into four Modules: 1, definitions (15 statements); 2, conservative and medical management (21 statements); 3, endoscopy (14 statements); and 4, metabolic bariatric surgery (86 statements). These 136 statements spanning Modules 1–4 then were balanced by the Delphi expert to minimize the risk that the survey instrument itself might induce bias by using response options other than agree/disagree, converting as many statements as possible into non-judgmental statements (neither favorable nor unfavorable to the concept presented), balancing all remaining statements to ensure roughly equal numbers of favorable and non-favorable statements, and adjusting the response options so favorable options were equally distributed in response order. The survey was then reviewed by all advisory group members for a pilot test and final editing. Consensus was defined as ≥ 70% inter-voter agreement, and a valid vote as voter participation ≥ 80%. Voting on statements specifically addressing the technical aspects of MBS was restricted to clinicians with sufficient expertise on the issue.

Given the critical importance of establishing consensus on definitions prior to progressing to statements on treatment specifics, the advisory panel dedicated Day #1 of the conference to Module 1, with open discussion prior to voting, and Day #2 to Modules 2–4, using published Delphi survey guidelines [19]. The current literature and level of evidence on all statements were presented by the specific experts to the whole consensus group prior to open discussion and voting. The 2-day conference in-person discussion and voting and online voting procedures are depicted in detail in both text and schematic form in online Supplement 1.

### Data Analysis

Data analysis was completed between rounds to identify statements with 70% consensus reached or not reached, and whether adequate voter participation had been achieved. Statements not achieving either 70% consensus or 80% eligible voter participation were included in the next round of voting.

## Results

The 43-member expert panel included 17 from Europe, 13 from North America, six from Latin America, 5 from the Middle East and Northern Africa, and 2 from Asia-Oceania. There were 26 bariatric surgeons including 2 pediatric bariatric surgeons, among whom 11 also performed endoscopic bariatric procedures. The remaining expert panel members were four endoscopists, eight endocrinologists, one internist, one pediatrician, two nutritionists, and two counsellors (psychology, exercise).

The final survey had 136 statements, with one deleted from Module 4 because it duplicated an earlier statement. Forty-seven statements underwent change after Round 1 to such a degree that the Round 1 results were considered invalid and considered new statements in Round 2A.

Since we considered it critical to achieve consensus on all the definition statements (Module 1), and considerable discussion occurred to achieve this, consensus was reached on all 15 at a mean level of 90.1% consensus. There was great variability over Modules 2–4 on both the percentage of statements on which consensus was reached (50.0–95.2%) and the overall mean level of consensus achieved (66.7–86.9%). Consensus was reached on 68/85 MBS statements with a mean level of consensus of 79.6% (Supplementary Table A). Among the 38 statements that required two rounds of voting, consensus only was reached on 13: both statements on definitions and 11 of the 28 MBS statements. All seven statements on endoscopy failing to achieve consensus in Round 1 also failed in Round 2 (Supplementary Table B).

### Module 1—Definitions

Module 1 results for 15 definitions statements are listed in Table 1. A suboptimal initial response was unanimously defined as inadequate weight loss or an unusually modest improvement in a clinically significant obesity complication, and 39/40 experts agreed that the severity of a suboptimal response should guide treatment. Similar to suboptimal response, late postoperative deterioration was defined as recurrent weight gain or worsening of a significant obesity complication. Baseline weight in patients who undergo MBS was defined as that measured before starting preoperative weight reduction. With respect to the use of re-operative MBS to address either a suboptimal initial response, later clinical deterioration, or adverse events, experts agreed that surgical or endoscopic procedures to convert to a new type of MBS (conversion surgery) or to reestablish normal anatomy (reversal surgery) in major adverse events should be clearly distinguished and considered separately from procedures to modify or revise a previous operation (modification or revision surgery). Experts agreed that the presumed mechanism of action should not be used to describe MBS procedures, which instead should be labelled by the anatomical changes made. There was strong consensus on omitting demeaning terms like “super-obesity” to describe a BMI > 50 kg/m^2^ and on replacing such terms with a BMI-based classification system (e.g., class IV, BMI 50-60 kg/m^2^; class V, BMI > 60 kg/m^2^).

**Table 1:** Module 1—reporting definitions and standards

Statements		*N*	Rounds	Most common	Percentage
			required	selection	consensus
Reporting definitions					
1	A suboptimal initial response to metabolic/bariatric surgery is demonstrated either by inadequate weight loss OR by an unusually modest improvement in a significant obesity complication	41	1	Agree	100.0%
2	A late post-operative clinical deterioration is demonstrated either by recurrent weight gain OR by worsening of a significant obesity complication that occurs after an initially adequate post-operative clinical response	38	1	Agree	97.4%
3	The degree to which the clinical response to metabolic/bariatric surgery is suboptimal or there is a late post-operative clinical deterioration can vary widely from patient to patient. The severity of the suboptimal response should guide clinical treatment	40	1	Agree	97.5%
4	The baseline weight for assessing weight loss after MBS should be a weight determined before starting preoperative weight reduction	43	3	Agree	95.3%
5	In patients who have been treated with AOM before undergoing MBS, who STOP it at the time of or shortly after surgery, the baseline weight for assessing the effect of surgery on bodyweight should generally be a weight determined BEFORE the AOM was started	43	2	Agree	95.3%
6	In patients who have been treated with AOM before undergoing MBS and CONTINUE this medication post-op., the baseline weight used to assess the effect of surgery on body weight should generally be measured on the day of surgery	44	3	Agree	88.4%
7	The initial surgical weight loss (defined as maximum weight loss within the first 2 years after MBS) should be determined in a manner that excludes any post-plateau weight loss caused by adding AOM, any endoscopic intervention, or any calorie-restricted diet	38	2	Agree	84.2%
8*	Surgical or endoscopic procedures to convert to a new type of metabolic/bariatric operation (conversion surgery) and those to re-establish normal anatomy (reversal surgery) should be clearly distinguished and considered separately from procedures to modify or enhance the effects of a previous operation (revision or modification surgery)	40	1	Agree	97.5%
9*	Modification or revision procedures are typically designed to optimize the effectiveness of previous operations, while conversion procedures most commonly introduce additional mechanisms of therapeutic action	40	1	Agree	95.0%
10	The term “obesity complication” mostly describes diseases, conditions, and symptoms for which there is published evidence that obesity is a contributing cause or exacerbating factor. When such a causative relationship has not been established or accepted, the associated disorder is more accurately labelled an obesity comorbidity	41	2	Agree	80.5%
11	When considering the effects of MBS on intestinal nutrient absorption, diminished absorption (hypo-absorption or malabsorption) of micronutrients should be clearly distinguished from the hypo-absorption or malabsorption of macronutrients or ingested calories	42	2	Agree	85.7%
12	Characterization of the absorptive effects of an MBS procedure should not be used to imply that these effects are the mechanisms of action of weight loss associated with the operation. It is preferable to describe such procedures by their anatomical features (e.g., “bypass,” “diversion,” or more generally, “gastrointestinal”) rather than by their inferred mechanism of action	41	2	Agree	95.1%
13	Characterization of the changes in the physical structure of the gut produced by an MBS procedure – including the size & shape of GI segments or anastomoses – should not be used to imply that these changes “restrict” food intake as a mechanism of associated weight loss. It is preferable to describe such procedures by their anatomical features (e.g., “gastrectomy,” “banding” or, more generally, “gastric”) rather than by their inferred mechanism of action	41	2	Agree	95.1%
Reporting standards					
14	In general, a suboptimal initial clinical response to MBS is demonstrated either by total body weight or BMI loss of less than 20% OR by inadequate improvement in an obesity complication that was a significant indication for surgery	40	1	Agree	85.0%
15	In general, a late post-operative clinical deterioration after MBS is demonstrated either by recurrent weight gain of more than 30% of the initial surgical weight loss OR by worsening of an obesity complication that was a significant indication for surgery	39	1	Agree	71.8%

*N*, number of voters in deciding round; *MBS*, metabolic bariatric surgery; *AOM*, anti-obesity medication; *GI*, gastrointestinal; *BMI*, body mass index. All statements reached at least 70% consensus

*Instructions provided to the experts before they voted on statements 8 and 9 were to first read through both sentences to provide context and then vote on each statement separately

### Modules 2–4

Results for Modules 2–4 are summarized in Tables 2, 3, 4, 5, and 6, with all statements pertaining to conservative and medical management in Table 2; endoscopy, Table 3; specific MBS procedures, Table 4; special circumstances related to patient BMI and age (≤ 18, ≥ 65), Table 5; and obesity complications, Table 6.

**Table 2:**
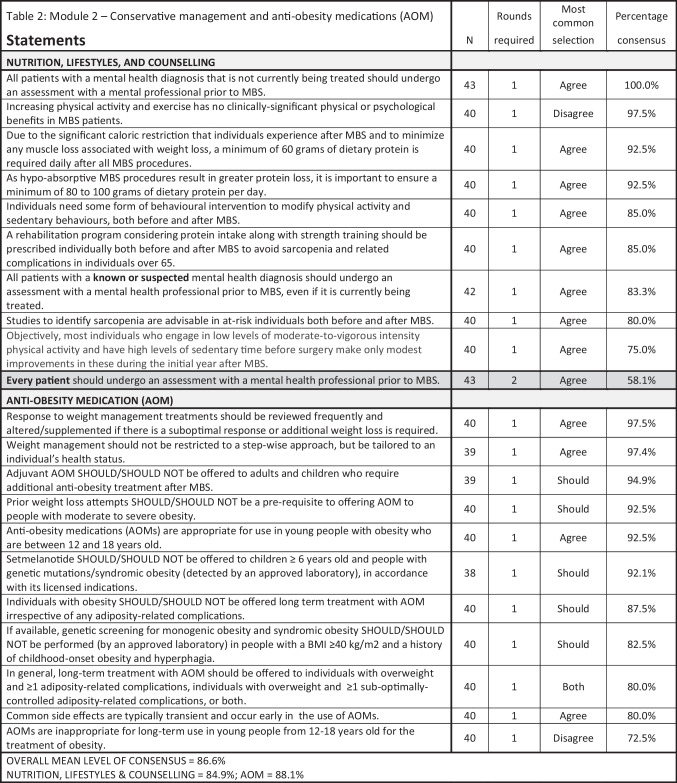
Module 2—conservative management and anti-obesity medications (AOM). N, number of voters in deciding round; MBS, metabolic and bariatric surgery; AOM, anti-obesity medication Shaded cells indicate non-consensus

**Table 3:**
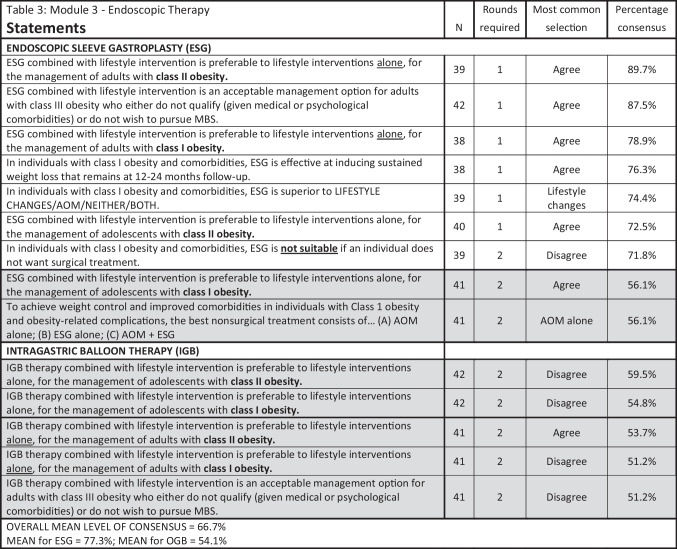
Module 3—endoscopic therapy. N, number of voters in deciding round; ESG, endoscopic sleeve gastroplasty; IGB, intra-gastric balloon; MBS, metabolic and bariatric surgery; AOM, anti-obesity medication. Shaded cells indicate non-consensus

**Table 4:**
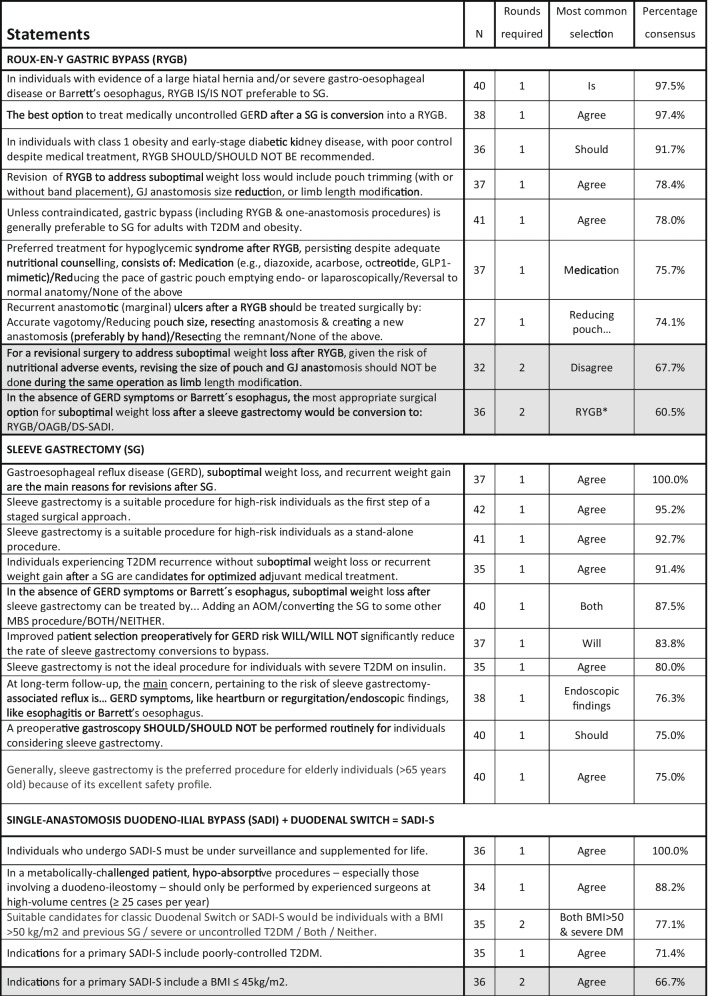

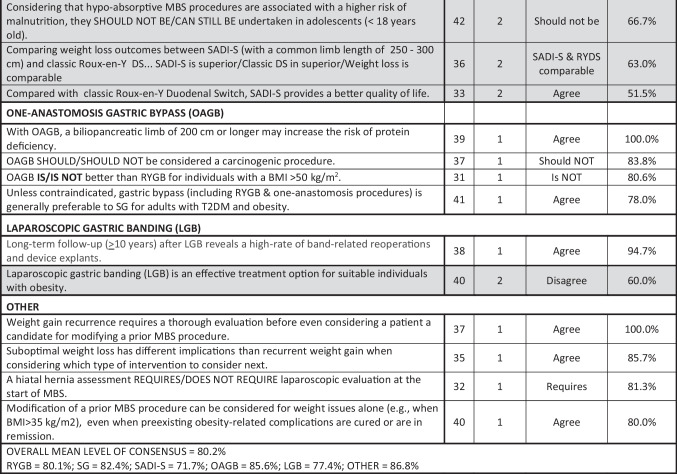
Module 4A—surgical procedures. N, number of voters in deciding round; AOM, anti-obesity medication; BMI, body mass index; DS, duodenal switch; GERD, gastro-esophageal reflux disease; GJ, gastro-jejunal; MBS, metabolic and bariatric surgery; OAGB, one-anastomosis gastric bypass; RYGB, Roux-en-Y gastric bypass; RY-DS, Roux-en-Y duodenal switch; SADI, single-anastomosis duodenal-ileal bypass; SADI-S, SADI with sleeve gastrectomy; LGB, laparoscopic gastric banding; T2DM, type 2 diabetes mellitus; VTE, venous thromboembolism. Shaded cells indicate non-consensus.

**Table 5:**
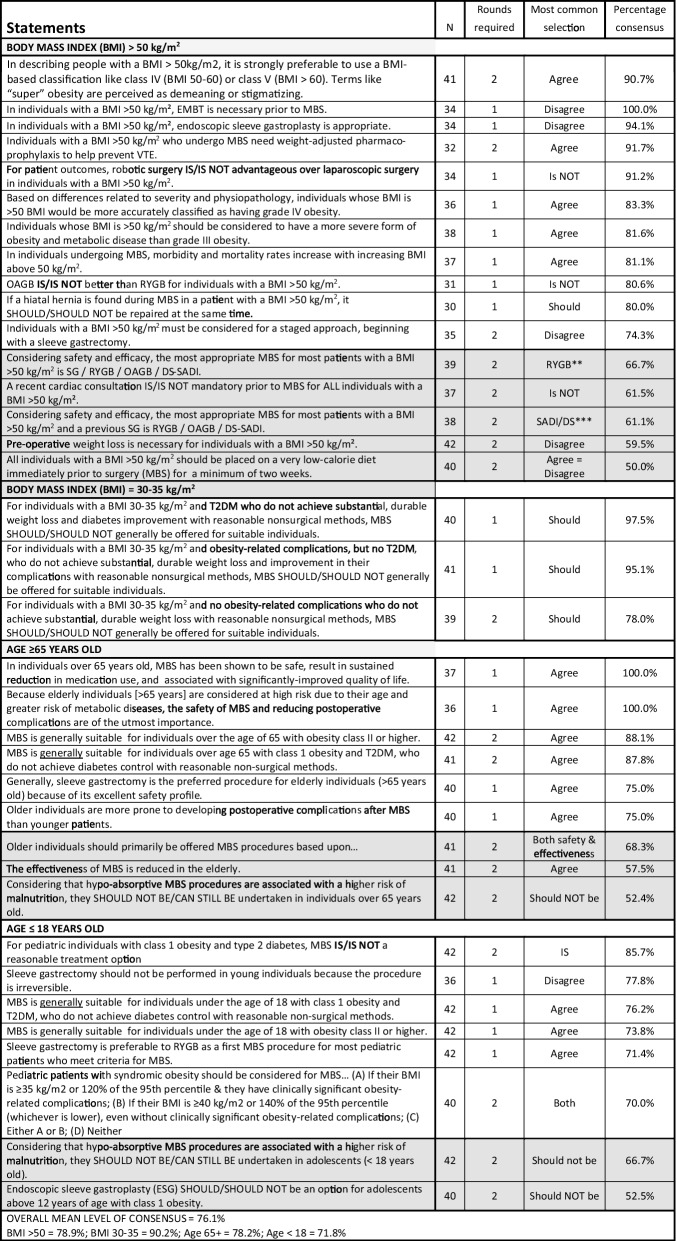
Module 4B—special circumstances by body mass index and age. N, number of voters in deciding round; AOM, anti-obesity medication; BMI, body mass index; DS, duodenal switch; ESG, endoscopic sleeve gastroplasty; GERD, gastro-esophageal reflux disease; HTN, hypertension; MBS, metabolic and bariatric surgery; OAGB, one-anastomosis gastric bypass; RYGB, Roux-en-Y gastric bypass; RY-DS, Roux-en-Y duodenal switch; SADI, single-anastomosis duodenal-ileal bypass; SADI-DS, SADI with duodenal switch; SADI-S, SADI with sleeve gastrectomy; T2DM, type 2 diabetes mellitus. Shaded cells indicate non-consensus. *Other procedures with percentage votes = SADI/DS 26.3%, OAGB 13.2%. **Other procedures with percentage votes = SADI/DS 12.8%, SG 10.3%, OAGB 10.3%. ***Other procedures with percentage votes = RYGB 25.0%, OAGB 13.9%

**Table 6:**
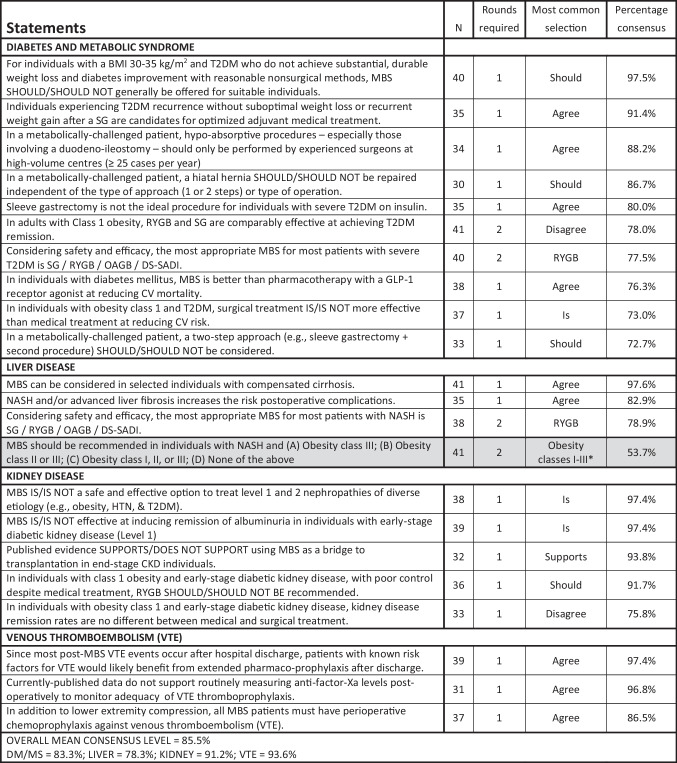
Module 4C—complications of obesity. N, number of voters in deciding round; AOM, anti-obesity medication; BMI, body mass index; CV, cardiovascular; DS, duodenal switch; ESG, endoscopic sleeve gastroplasty; GERD, gastro-esophageal reflux disease; GLP, glucagon-like peptide; HTN, hypertension; MBS, metabolic and bariatric surgery; NASH, non-alcoholic steatohepatitis; OAGB, one-anastomosis gastric bypass; RYGB, Roux-en-Y gastric bypass; RY-DS, Roux-en-Y duodenal switch; SADI, single-anastomosis duodenal-ileal bypass; SADI-DS, SADI with duodenal switch; SADI-S, SADI with sleeve gastrectomy; T2DM, type 2 diabetes mellitus; VTE, venous thromboembolism. Shaded cells indicate non-consensus. *Other selections with percentage of votes = class II and III obesity 31.7%, class III obesity 7.3%, not I, II or III 7.3%

## Discussion

In this IFSO-sponsored expert consensus survey, we achieved unanimous consensus on all reporting definitions and reporting standards. This was accomplished by a multidisciplinary expert group, paving the way to implementing these definitions in clinical practice and research within both the surgical and medical communities. The other focus of this survey was to address MBS topics where long-term evidence-based data remain insufficient. It serves as a next step following the joint IFSO and World Gastroenterology Organization (WGO) Delphi survey conducted in the spring of 2022 examining all non-surgical approaches to obesity management, including patient assessment and preparation for MBS.

Our first major objective, which we considered vital to constructing any MBS guidelines, was to achieve consensus on essential definitions pertaining to surgical management of obesity—including definitions for weight loss attributable to MBS (versus concomitant therapies), baseline weight, suboptimal initial clinical response, recurrent weight gain, and distinguishing conversion from revision surgery. This we achieved, through considerable discussion and major modifications to original statements. As weight loss is the driving force behind positive outcomes after MBS, all statements on weight loss were written to standardize reporting, starting from baseline weight to nadir weight loss 2 years after surgery to recurrent weight gain. However, as the outcome of MBS is a composite endpoint, obesity comorbidities and complications are included in the definitions.

The uniform standardized use of these definitions paves the way to enhancing comparability of the reoperations and their indications within the field of obesity treatment and MBS. The current literature mixes revisional surgery and conversion surgery terms, not to mention the variability of indications for reoperation. The firm consensus of our experts was to clearly differentiate these terms as modification or revision procedures are typically designed to optimize the effectiveness of previous operations, while conversion procedures most commonly introduce additional mechanisms of therapeutic action. For the accurate categorization of different procedures, the consensus was reached that MBS procedures should be labelled by the anatomical changes made intraoperatively (e.g., gastric bypass), rather than by presumed mechanisms of action (e.g., restriction, malabsorption, or hypo-absorption). The rationale was that the mechanisms by which MBS procedures work are complex, frequently multi-faceted (e.g., involving other factors like hormonal and neuronal effects), and often incompletely understood.

The biological basis of obesity and the response to MBS underscore the importance of recognizing that a suboptimal clinical response rarely reflects either substandard surgical skill or technique. Similarly, it is rarely caused by noncompliance or other aberrant or inadequate behavior by the patient. Thus, the language we use to describe less robust clinical outcomes must avoid being judgemental, ascribing blame, or drawing unproven, causal inferences. Thus, by consensus, we recommend that less than ideal weight loss or clinical improvement after MBS be described as a “suboptimal clinical response” or “suboptimal weight loss,” rather than “non-response” or an “inadequate” response to treatment. Similarly, consensus was reached on using “recurrent weight gain” for those who experience significant weight gain after initial postoperative weight loss.

Within the normal distribution of weight loss response to MBS, there is no specific magnitude of weight loss that clearly differentiates between treatment success and suboptimal response. There is some evidence that 20% of total body weight loss is associated with reduced cardiovascular risk [20, 21], so many clinicians and investigators have used this criterion to assess clinical responses to MBS. It is recognized that the magnitude of weight loss has widely different clinical effects in different patients, and a categorical definition of weight loss should not be used as the single determinant of the need for additional clinical intervention. Through this Delphi process, unanimous consensus was achieved on using the following reporting standards for “suboptimal initial clinical response” as initial total body weight loss < 20% or inadequate improvement in an obesity complication that was a significant indication for surgery and “recurrent weight gain” as recurrent weight gain > 30% or worsening of an obesity complication that was a significant indication for surgery. Given the different effectiveness of each MBS procedures [13, 14] and variable effects in different populations, these criteria should be applied to individual patients combined with expert clinical judgement.

As in the previous IFSO/WGO survey, ensuring adequate nutritional supplementation was strongly agreed upon, as was acceptance of the roles of AOM spanning virtually all clinical scenarios: as first-line therapy, in young and old patients, before and after MBS, and for both short-term and long-term use. Like other chronic diseases, the treatment of obesity should follow the principles of chronic disease management with a combination of treatment options. For obesity, combination therapies failed to progress in the past due to the lack of effective AOMs. With the increased availability of current available potent AOMs and in the pipeline, the practice of combination therapy will likely increase as MBS and AOMs can work in synergy. Conversely, amongst our experts panel, considerable disagreement/non-consensus was observed regarding the role of metabolic bariatric endoscopy due to a lack of strong scientific evidence in the literature, though the use of ESG, combined with lifestyle interventions, was consistently supported in patients with class I and II obesity, with or without obesity-related complications, and with class III obesity who either do not qualify for or choose not to pursue MBS.

Numerous studies have demonstrated that MBS is significantly more effective than dietary and lifestyle changes alone at inducing weight loss, reducing complications, comorbidities, and mortality, and improving patients’ overall quality of life [6, 8, 10, 11, 22–24]. Such reductions in complications and comorbidities include improvements in existing conditions and their prevention, including the prevention of various cardiometabolic diseases and cancers demonstrated also in multiple meta-analyses [25–34]. But questions persist as to when MBS might be contraindicated and which procedure to select in different situations.

Worldwide, SG has become the most common MBS procedure performed, and our experts agreed that it is the most suitable choice for high-risk patients, pediatric patients, and seniors > 65. However, our experts also agreed that SG is less suitable in patients with certain obesity complications such as poorly controlled T2DM, GERD, or NASH. It was also the most commonly selected procedure for patients with a BMI ≥ 50 kg/m^2^ (by 66.7%), though no consensus was achieved. Voting on biliopancreatic diversion with duodenal switch (BPD-DS) and sleeve gastrectomy single-anastomosis duodenoileostomy (SADI), the consensus was that suitable candidates include patients with a BMI > 50 kg/m^2^ and with severe or uncontrolled diabetes. However, those who undergo SADI-S will require surveillance and nutritional supplements for life. Voting on one-anastomosis gastric bypass (OAGB), there was 100% consensus that a biliopancreatic limb ≥ 200 cm increases the risk of protein deficiency and that, unless otherwise contraindicated, both RYGB and OAGB are generally preferable to SG for adults with both T2DM and obesity.

Every expert consensus survey has the potential for bias, given that clinicians considered experts in a particular practice must utilize it to be considered experts. We tried to minimize such bias in numerous ways, including seeking the opinions of 17 multi-disciplinary non-surgeons with expertise in obesity management; by including experts from every continent; by taking several steps, like statement balancing, to minimize any bias inherent in the survey itself; and by assistance of an internationally recognized expert in Delphi surveys.

We acknowledge that consensus surveys rely on opinions, rather than experimentally generated data and represent level V evidence. On the other hand, our experts were all widely renowned experts in obesity management, most contributing extensively to obesity research, and were, thus, both highly familiar with and qualified to interpret their expansive knowledge of the literature. Ultimately, these consensus results will be used as an adjunct to a thorough literature review to guide clinical practice and assist in creating an algorithm to aid clinicians in their decisions when treating patients with obesity.

### Supplementary Information

Below is the link to the electronic supplementary material.

Supplementary file1 (MOV 11854 KB)

Supplementary file2 (DOCX 557 KB)
