# Supplementary material for: IFSO Consensus on Definitions and Clinical Practice Guidelines for Obesity Management—an International Delphi Study
Source: Obes Surg. 2023 Nov 24;34(1):30–42. doi: 10.1007/s11695-023-06913-8 (PMC10781804; doi:10.1007/s11695-023-06913-8)
Supplement: Supplementary file 2 — Supplementary file2 (DOCX 557 KB) [file 11695_2023_6913_MOESM2_ESM.docx]

Online supplementary file 1

SUPPLEMENTARY MATERIALS

**DETAILED DESCRIPTION OF SURVEY METHODS**

A three-round Delphi survey of 43 intercontinental, interdisciplinary experts in obesity management was conducted, beginning with in-person voting over two days in Hamburg, Germany, from March 9-10, 2023, followed by discussion, followed by two rounds of online voting. The expert panel included surgeons, endoscopists, internists, dieticians, and councillors with obesity management expertise. To be considered for the expert panel, clinicians had to (a) have obesity management as a major focus of their practice, (b) be considered experts by IFSO, (c) have ≥10 years managing patients with obesity, (d) be fluent in both spoken and written English, and (e) be willing to attend, either in person or on Zoom, a two-day conference in Hamburg for expert lectures on published literature, open discussion, and a Delphi survey.

**SURVEY DEVELOPMENT**

In January 2023, each expert who agreed to participate was asked to contribute 3-5 statements/questions within their field for consideration by a core advisory group comprised of the primary authors and an MD-PhD level expert in Delphi surveys (KPW). This yielded >300 submitted statements. Over six virtual meetings of the core advisory group, these were these were pared down to 136 statements subdivided into four Modules: **1-Definitions** (15 statements); **2-Conservative and medical management** (21 statements); **3-Endoscopy** (14 statements); and **4- Metabolic bariatric surgery** (86 statements). Note that IFSO and the World Gastroenterology Organization (WGO) had just completed a 94-expert Delphi on the non-surgical management of obesity. These 136 statements spanning Modules 1-4 then were balanced by the Delphi expert to minimize the risk that the survey instrument itself might induce bias by using response options other than AGREE/DISAGREE; converting as many statements as possible into non-judgemental statements (neither favorable nor unfavorable to the concept presented); balancing all remaining statements to ensure roughly equal numbers of favorable and non-favorable statements; and adjusting the response options so favorable options were equally distributed in response order. The survey then was reviewed by all advisory group members for a pilot test and final editing. Consensus was defined as ≥70% inter-voter agreement, and a valid vote as voter participation ≥80%. Voting on statements specifically addressing the technical aspects of MBS was restricted to physicians.

Given the critical importance of establishing consensus on definitions prior to progressing to statements on treatment specifics, the advisory panel dedicated Day #1 of the conference to Module 1, with open discussion prior to voting, and Day #2 to Modules 2-4, using published Delphi survey guidelines[24].

**CONFERENCE DAY 1 - MODULE 1: DEFINITIONS**

Given the critical importance of establishing consensus on definitions, prolonged discussion of each statement was permitted over four hours, during which only one round of voting was initiated. Since subsequent discussion revealed considerable non-consensus on several definitions, largely attributed to concerns over the wording of statements, a decision was made to perform a second and, if needed, further rounds of voting online, permitting experts to provide comments with each statement in Round 2, which would be reviewed by the advisory board to consider further statement adjustments prior to, if necessary, a third round of voting.

**CONFERENCE DAY 2**

During Round 1 voting on Modules 2-6, two minutes were allotted per statement, only permitting questions to clarify a statement’s meaning prior to voting. Any discussion on statement contents or wording was restricted to after Round 1, as explained below. During voting, each statement was projected onto two screens to ensure adequate visibility. For each statement, voting was permitted for a maximum of 30 seconds before progressing to the next statement.

As on Day 1, discussion of Round 1 results revealed considerable concerns pertaining to the wording of certain statements. For this reason, as with Module 1, Round 2 voting was converted to an online format, affording the core committee time to consider the concerns expressed – both during the meeting and via email communication afterwards – to permit potential rewording of contentious statements before Round 2.

**ONLINE VOTING**

Once a final edition of the second-round survey was developed and approved by the advisory committee – encompassing statements in Modules 1-4 on which no consensus was reached in Round 1 – an email was sent to all 43 experts containing instructions and a link to the survey on the online platform Survey Monkey. Voting was permitted for up to 14 days after that email was sent, with email reminders sent to non-responders at 7 and 10 days, followed by a telephone call. After 14 days, further voting was blocked. Any statements that had undergone major revisions from their Round 1 version, based upon expert discussion, were considered NEW statements in Round 2A, with a third round of voting (Round 2B) added for such statements for which no consensus was reached in Round 2A. Steps to Rounds 1, 2A, and 2B are depicted in Figure A, immediately below.

FIGURE 1: Schematic of the Delphi survey process


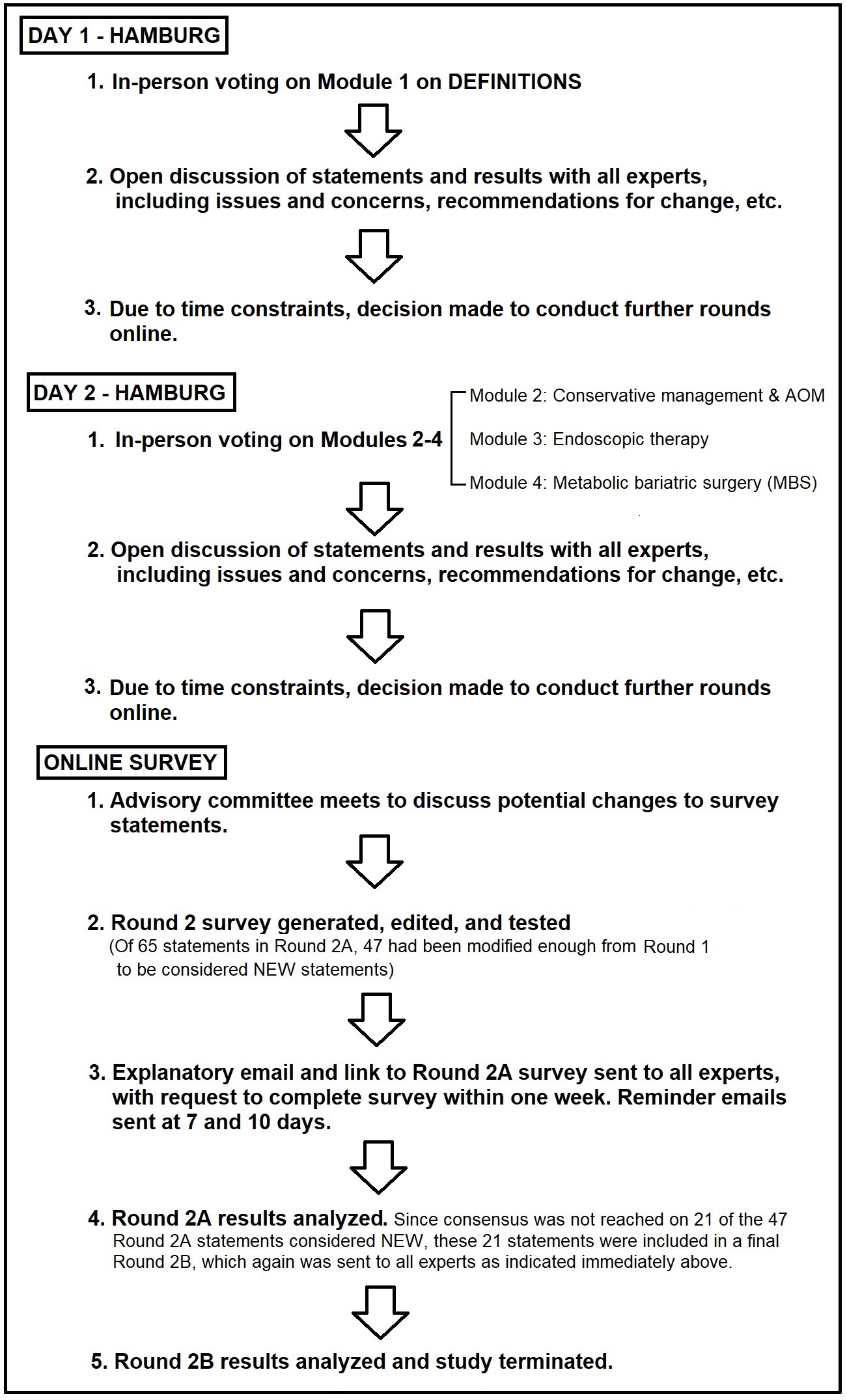


**DATA ANALYSIS**

Data analysis was completed between rounds to identify statements with 70% consensus reached or not reached, and whether adequate voter participation had been achieved. Statements not achieving either 70% consensus or 80% eligible-voter participation were included in the next round of voting.

Supplementary Table A: Overall Delphi results

| **Statements** | **N of statements** | **% =** |
| --- | --- | --- |
| Total number of statements | 135 | 100% |
| Consensus reached | 110 | 81.5% |
| No consensus reached | 25 | 18.5% |
| Consensus reached in 1st round (among 106 statements reaching consensus) | 97 | 88.1% |
| Consensus reached in 2nd round (among 106 statements reaching consensus) | 13 | 11.9% |
| Consensus NOT reached in 2nd round (among 38 statements voted on) | 25 | 65.8% |
| **TOTAL** | **135** | **100%** |
| % statements consensus reached - Module 1: Definitions | 15/15 | 100% |
| % statements consensus reached - Module 2: Nutrition/ Psychology/Lifestyle/Medication | 20/21 | 95.2% |
| % statements consensus reached - Module 3: Endoscopic therapy | 7/14 | 50.0% |
| % statements consensus reached - Module 4-1: Bariatric surgery - General principles | 26/32 | 81.3% |
| % statements consensus reached - Module 5: Bariatric surgery - BMI and age | 23/33 | 69.7% |
| % statements consensus reached - Module 6: Bariatric surgery - Comorbidities of obesity | 19/20 | 95.0% |
| **TOTAL** | **110/135** | **80.5%** |
| 100% consensus reached (among 110 statements reaching consensus) | 9 | 8.2% |
| 90-99% consensus reached (among 110 statements reaching consensus) | 34 | 30.9% |
| 80-89% consensus reached (among 110 statements reaching consensus) | 34 | 30.9% |
| 70-79% consensus reached (among 110 statements reaching consensus) | 33 | 30.0% |
| **TOTAL** | **109** | **100%** |
| Statements agreed with (total) | 76 | 82.6% |
| Statements disagreed with (total) | 15 | 16.3% |
| Statements with agree = disagree | 1 | 1.1% |
| **TOTAL** | **92** | **100%** |
| Statements agreed with (consensus) | 69 | 88.5% |
| Statements disagreed with (consensus) | 9 | 11.5% |
| Statements with agree = disagree | 0 | 0.0% |
| **TOTAL** | **78** | **100%** |
| Statements worded favorably | 34 | 25.2% |
| Statements worded unfavorably | 31 | 23.0% |
| Non-judgemental statements | 70 | 51.9% |
| **TOTAL** | **135** | **100.0%** |
| Average consensus - Module 1: Definitions | 90.9% |  |
| Average consensus - Module 2: Nutrition/Psychology/Lifestyles/Medication | 86.6% |  |
| Average consensus - Module 3: Endoscopic therapy | 50.0% |  |
| Average consensus - Module 4: Bariatric surgery - General | 82.5% |  |
| Average consensus - Module 5: Bariatric surgery - BMI and age | 76.1% |  |
| Average consensus - Module 6: Bariatric surgery - Comorbidities of obesity | 80.6% |  |
| **Average consensus - OVERALL** | 80.6% |  |
| Minimum/maximum consensus on any statement | 50.0%/100% |  |
| Min. when consensus reached | 70% |  |

Supplementary Table B: Statements requiring two rounds of voting

| Statement | Number of voters in Round 1 | % consensus in Round 1 | Most common choice | Number of voters in Round 2 | % consensus in Round 2 | Most common choice |
| --- | --- | --- | --- | --- | --- | --- |
| **Module 1: Definitions** | | | | | | |
| The baseline weight for assessing weight loss after MBS should be a weight determined before starting preoperative weight reduction. | 42 | 64.3% | Agree | 43 | 95.3% | Agree |
| In patients who have been treated with AOM before undergoing MBS and CONTINUE this medication post-op., the baseline weight used to assess the effect of surgery on body weight should generally be measured on the day of surgery. | 42 | 64.3% | Agree | 43 | 88.4% | Agree |
| **Module 2: Non-Surgical/Non-Endoscopic Management** | | | | | | |
| **Every patient** should undergo an assessment with a mental health professional prior to MBS. | 43 | 65.1% | Agree | 43 | 58.1% | Agree |
| **Module 3: Endoscopic Metabolic and Bariatric Therapy (EMBT)** | | | | | | |
| IGB therapy combined with lifestyle intervention is preferable to lifestyle interventions alone, for the management of adolescents with **class II obesity.** | 42 | 52.4% | Disagree | 42 | 59.5% | Disagree |
| ESG combined with lifestyle intervention is preferable to lifestyle interventions alone, for the management of adolescents with **class I obesity.** | 38 | 52.6% | Agree | 41 | 56.1% | Agree |
| To achieve weight control and improved comorbidities in individuals with Class 1 obesity and obesity-related complications, the best nonsurgical treatment consists of… (A) AOM alone; (B) ESG alone; (C) AOM + ESG | 31 | 58.1% | AOM alone | 41 | 56.1% | AOM alone |
| IGB therapy combined with lifestyle intervention is preferable to lifestyle interventions alone, for the management of adolescents with **class I obesity.** | 40 | 50.0% | A = D | 42 | 54.8% | Disagree |
| IGB therapy combined with lifestyle intervention is preferable to lifestyle interventions alone, for the management of adults with **class II obesity.** | 42 | 52.4% | Agree | 41 | 53.7% | Agree |
| IGB therapy combined with lifestyle intervention is preferable to lifestyle interventions alone, for the management of adults with **class I obesity.** | 41 | 56.1% | Agree | 41 | 52.1% | Disagree |
| IGB therapy combined with lifestyle intervention is an acceptable management option for adults with class III obesity who either do not qualify (given medical or psychological comorbidities) or do not wish to pursue MBS. | 41 | 51.2% | Disagree | 41 | 51.2% | Disagree |
| **Module 4: Metabolic and Bariatric Surgery Procedures** | | | | | | |
| For individuals with a BMI 30-35 kg/m2 and no obesity-related complications who do not achieve substantial, durable weight loss with reasonable nonsurgical methods, MBS SHOULD/SHOULD NOT generally be offered for suitable individuals. | 39 | 66.7% | Should | 41 | 78.0% | Should |
| Recurrent anastomotic (marginal) ulcers after a RYGB should be treated surgically by: Accurate vagotomy/Reducing pouch size, resecting anastomosis & creating a new anastomosis (preferably by hand)/Resecting the remnant/None of the above. | 30 | 63.3% | Reduce pouch… | 27 | 74.1% | Reduce pouch… |
| Indications for a primary SADI-S include a BMI ≤ 45kg/m2. | 36 | 58.3% | Agree | 36 | 66.7% | Agree |
| Laparoscopic gastric banding (LGB*) is an effective treatment option for suitable individuals with obesity. | 42 | 59.5% | Disagree | 40 | 60.0% | Disagree |
| Compared with classic Roux-en-Y Duodenal Switch, SADI-S provides a better quality of life. | 32 | 53.1% | Agree | 33 | 51.5% | Agree |
| For a revisional surgery to address suboptimal weight loss after RYGB, given the risk of nutritional adverse events, revising the size of pouch and GJ anastomosis should NOT be done during the same operation as limb length modification. | 32 | 56.3% | Disagree | 31 | 67.7% | Disagree |
| Comparing weight loss outcomes between SADI-S (with a common limb length of 250 - 300 cm) and classic Roux-en-Y DS... SADI-S is superior/Classic DS in superior/Weight loss is comparable | 35 | 65.7% | Comparable | 27 | 63.0% | Comparable |
| In the absence of GERD symptoms or Barrett´s esophagus, the most appropriate surgical option for suboptimal weight loss after a sleeve gastrectomy would be conversion to: RYGB/OAGB/DS-SADI. | 36 | 44.4% | RYGB | 38 | 60.5% | RYGB |
| **Module 4B-1: Body mass index** | | | | | | |
| Individuals with a BMI >50 kg/m^2^ who undergo MBS need weight-adjusted pharmaco-prophylaxis to help prevent VTE. | 32 | 59.4% | Agree | 32 | 91.7% | Agree |
| Suitable candidates for classic Duodenal Switch or SADI-S would be individuals with a BMI >50 kg/m^2^ and previous SG / severe or uncontrolled T2DM / Both / Neither. | 36 | 69.4% | Both | 37 | 77.1% | Both |
| Considering safety and efficacy, the most appropriate MBS for most patients with a BMI >50 kg/m^2^ is SG / RYGB / OAGB / DS-SADI. | 40 | 50.0% | RYGB | 39 | 66.7% | RYGB |
| A recent cardiac consultation IS/IS NOT mandatory prior to MBS for ALL individuals with a BMI >50 kg/m². | 37 | 54.1% | Is NOT | 39 | 61.5% | Is NOT |
| Considering safety and efficacy, the most appropriate MBS for most patients with a BMI >50 kg/m^2^ and a previous SG is RYGB / OAGB / DS-SADI. | 38 | 50.0% | DS-SADI | 36 | 61.1% | DS-SADI |
| Pre-operative weight loss is necessary for individuals with a BMI >50 kg/m². | 35 | 54.3% | Disagree | 42 | 59.5% | Disagree |
| All individuals with a BMI >50 kg/m^2^ should be placed on a very low-calorie diet immediately prior to surgery ^16^ for a minimum of two weeks. | 34 | 61.8% | Disagree | 40 | 50.0% | A = D |
| **Module 4B-2: Age** (Adolescents and Patients ≥ 65 years old) | | | | | | |
| MBS is generally suitable  for individuals over the age of 65 with obesity class II or higher. | 40 | 65.0% | Selectively | 42 | 88.1% | Agree |
| MBS is generally suitable  for individuals over age 65 with class 1 obesity and T2DM, who do not achieve diabetes control with reasonable non-surgical methods. | 39 | 66.7% | Agree | 41 | 87.8% | Agree |
| For pediatric individuals with class 1 obesity and type 2 diabetes, MBS **IS/IS NOT** a reasonable treatment option | 40 | 67.5% | Is | 42 | 85.7% | Is |
| Pediatric patients with syndromic obesity should be considered for MBS… (A) If their BMI is ≥35 kg/m^2^ or 120% of the 95th percentile & they have clinically significant obesity-related complications; (B) If their BMI is ≥40 kg/m^2^ or 140% of the 95th percentile (whichever is lower), even without clinically significant obesity-related complications; (C) Either A or B; (D) Neither | 35 | 60.0% | Either A or B | 40 | 70.0% | Either A or B |
| Older individuals should primarily be offered MBS procedures based upon relative safety of the procedures, relative efficacy of the procedures, both, neither. | 39 | 53.8% | Safety | 41 | 68.3% | Both |
| Considering that hypo-absorptive MBS procedures are associated with a higher risk of malnutrition, they SHOULD NOT BE/CAN STILL BE undertaken in adolescents (< 18 years old). | 42 | 69.0% | Should NOT be | 42 | 66.7% | Should NOT be |
| The effectiveness of MBS is reduced in the elderly. | 41 | 51.2% | Agree | 40 | 57.5% | Agree |
| Endoscopic sleeve gastroplasty (ESG) SHOULD/SHOULD NOT be an option for adolescents above 12 years of age with class 1 obesity. | 34 | 50.0% | Should = Should not | 40 | 52.5% | Should NOT be |
| Considering that hypo-absorptive MBS procedures are associated with a higher risk of malnutrition, they SHOULD NOT BE/CAN STILL BE undertaken in individuals over 65 years old. | 42 | 59.5% | Should NOT be | 42 | 52.4% | Should NOT be |
| **Module 4C: Complications of obesity** | | | | | | |
| In adults with Class 1 obesity, RYGB and SG are comparably effective at achieving T2DM remission. | 34 | 67.6% | Disagree | 41 | 78.0% | Disagree |
| Considering safety and efficacy, the most appropriate MBS for most patients with severe T2DM is SG / RYGB / OAGB / DS-SADI. | 41 | 63.4% | RYGB | 40 | 77.5% | RYGB |
| Considering safety and efficacy, the most appropriate MBS for most patients with NASH is SG / RYGB / OAGB / DS-SADI. | 38 | 60.5% | RYGB | 38 | 78.9% | RYGB |
| MBS should be recommended in individuals with NASH and (A) Obesity class III; (B) Obesity class II or III; (C) Obesity class I, II, or III; (D) None of the above | 40 | 62.5% | Class I-III | 41 | 53.7% | Class I-III |

AOM = anti-obesity medication; BMI = body mass index; CV = cardiovascular; DS = duodenal switch; ESG = endoscopic sleeve gastroplasty; GERD = gastro-esophageal reflux disease; GLP = glucagon-like peptide; HTN = hypertension; MBS = metabolic and bariatric surgery; NASH = non-alcoholic steatohepatitis; OAGB = one-anastomosis gastric bypass; RYGB = Roux-en-Y gastric bypass; RY-DS = Roux-en-Y duodenal switch; SADI = single-anastomosis duodenal-ileal bypass; SADI-DS = SADI with duodenal switch; SADI-S = SADI with sleeve gastrectomy; T2DM = type 2 diabetes mellitus; VTE = venous thromboembolism. Shaded cells indicate non-consensus.
